# J-Net: Improved U-Net for Terahertz Image Super-Resolution

**DOI:** 10.3390/s24030932

**Published:** 2024-01-31

**Authors:** Woon-Ha Yeo, Seung-Hwan Jung, Seung Jae Oh, Inhee Maeng, Eui Su Lee, Han-Cheol Ryu

**Affiliations:** 1 Department of Artificial Intelligence Convergence, Sahmyook University, 815 Hwarang-ro, Nowon-gu, Seoul 01795, Republic of Korea; canal@syuin.ac.kr (W.-H.Y.); jshwan0828@naver.com (S.-H.J.); 2 Taean AI Industry Promotion Agency (TAIIPA), Taean County 32154, Republic of Korea; 3 YUHS-KRIBB Medical Convergence Research Institute, Yonsei University College of Medicine, 50-1 Yon-sei-ro, Seodaemun-gu, Seoul 03722, Republic of Korea; issac@yuhs.ac (S.J.O.); inheem@yonsei.ac.kr (I.M.); 4 Electronics and Telecommunications Research Institute (ETRI), Daejeon 34129, Republic of Korea

**Keywords:** terahertz images, image super-resolution, convolutional neural network (CNN), deep learning

## Abstract

Terahertz (THz) waves are electromagnetic waves in the 0.1 to 10 THz frequency range, and THz imaging is utilized in a range of applications, including security inspections, biomedical fields, and the non-destructive examination of materials. However, THz images have a low resolution due to the long wavelength of THz waves. Therefore, improving the resolution of THz images is a current hot research topic. We propose a novel network architecture called J-Net, which is an improved version of U-Net, to achieve THz image super-resolution. It employs simple baseline blocks which can extract low-resolution (LR) image features and learn the mapping of LR images to high-resolution (HR) images efficiently. All training was conducted using the DIV2K+Flickr2K dataset, and we employed the peak signal-to-noise ratio (PSNR) for quantitative comparison. In our comparisons with other THz image super-resolution methods, J-Net achieved a PSNR of 32.52 dB, surpassing other techniques by more than 1 dB. J-Net also demonstrates superior performance on real THz images compared to other methods. Experiments show that the proposed J-Net achieves a better PSNR and visual improvement compared with other THz image super-resolution methods.

## 1. Introduction

Terahertz (THz) imaging, operating within the 0.1 to 10 THz frequency range, is a rapidly evolving field with applications spanning from security inspections to biomedical and materials science. The unique characteristics of THz waves, bridging the gap between microwaves and infrared radiation, offer distinct advantages in these applications. Despite its potential, THz imaging is hindered by inherent limitations in resolution due to the longer wavelengths of THz waves compared to visible light. This results in images that are typically low in resolution, blurred, and noisy, making detailed analysis challenging.

The quest to enhance the resolution of THz images has led to a bifurcated approach: enhancing the imaging hardware and adopting super-resolution techniques. While hardware advancements can yield improvements, they often come with increased costs and complexity. Super-resolution methods, on the other hand, provide a more economical and flexible alternative, leveraging existing imaging systems to achieve high-resolution (HR) image reconstruction. Traditional methods, including deconvolution techniques like the Lucy–Richardson algorithm and various interpolation methods, have been instrumental in initial improvements. However, these approaches often fall short in recovering high-frequency details and handling noise variations [1,2,3].

The advent of deep learning and convolutional neural networks (CNNs) has revolutionized the field of image super-resolution. Pioneering techniques like the SRCNN [4], and subsequent advancements like the VDSR [5] and ESPCN [6] networks, have demonstrated superior performance in feature extraction and nonlinear modeling, significantly outperforming traditional methods. Numerous studies in THz imaging frequently adopt models originally proposed for optical image super-resolution in their research [7,8]. However, the direct application of these networks to THz imaging has been limited due to the unique degradation model of THz images, which includes blurring, downsampling, and noising with spatially variable characteristics.

In light of these challenges, our work introduces a novel network architecture, J-Net, an enhanced version of the U-Net framework [9]. U-Net has demonstrated exceptional performance in the field of image restoration [10,11,12,13,14]. Consequently, it is considered to be effectively applicable for image super-resolution as well, specifically tailored for THz image super-resolution. This is because image super-resolution is a subset of image restoration, focusing on enhancing resolution and detail, which is particularly relevant in the context of THz imaging where fine details are crucial. J-Net is designed to efficiently extract features from low-resolution THz images and learn the mapping to HR images. Unlike previous methods, J-Net is equipped with unique characteristics for super-resolving THz images, handling degradation models incorporating elements that address blurring, noise, and downsampling.

Through rigorous experimentation and comparison with established super-resolution methods, including the Lucy–Richardson deconvolution, Long et al. [7], and Ruan et al. [8], J-Net demonstrates superior accuracy and visual improvement in THz image super-resolution. This paper presents a comprehensive analysis of J-Net’s performance, showcasing its effectiveness through quantitative metrics such as peak signal-to-noise ratio (PSNR) on the DIV2K+Flickr2K dataset, along with qualitative assessments on real THz images.

## 2. Proposed Method

In this section, we begin by introducing the terahertz imaging system employed in this study, followed by an overview of the degradation model used to construct our training dataset. Subsequently, we provide a detailed explanation of the overall architecture of the proposed J-Net.

### 2.1. Terahertz Imaging System

**Figure 1:**
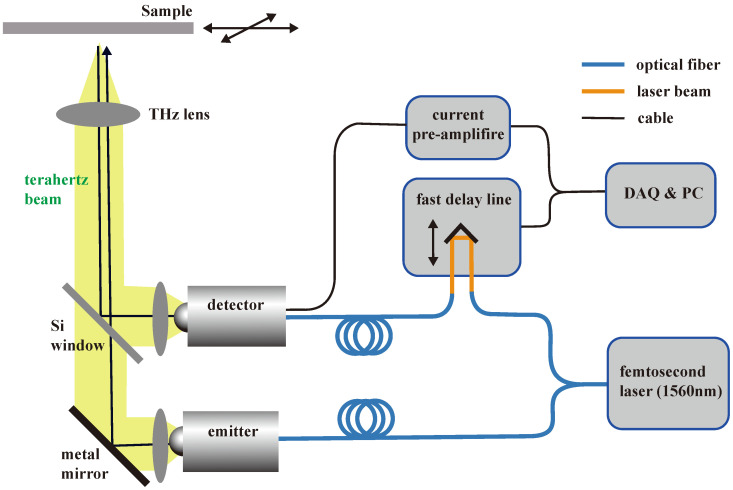
THz imaging system.

We used a reflection-mode THz imaging system to acquire images and THz waveforms, as shown in Figure 1. The THz pulse was obtained using a femtosecond laser and a photoconductive antenna. The femtosecond laser had a central wavelength of 1.5 μm and a pulse width of 80 fs. A fiber-coupled antenna (TERA15-TX-FC, Menlo Systems) was used as a photoconductive antenna for THz pulse generation, and another fiber-coupled dipole antenna (TERA15-RX-FC, Menlo Systems) was used to detect the THz signal. To rapidly acquire the THz signal, we used an ultra-fast scanner with frequency and time-width settings of 20 Hz and 30 ps, respectively.

The THz signal was amplified through a low-noise current preamplifier (SRS570, Stanford Research) and then digitized via a data acquisition board. The generated THz pulses were guided through a polymethylpentene (TPX) lens and plate mirrors. The TPX lens focused the THz signal onto the sample stage, and the signal reflected from the sample was guided through a silicon beam splitter, another TPX lens, and into the detector. In particular, all components of the system, except the sample, were placed within a dry-air chamber to avoid signal distortion due to water vapor absorption. The sample was placed in a 3 mm thick crystallized z-quartz window (subject to change). The time-domain signal obtained at each pixel was converted to the frequency domain via fast Fourier transform (FFT), thereby endowing each pixel with broadband THz wave characteristics. The sample was moved along the x and y axes to form a 2D image based on the response of the THz pulse signal. Each pixel’s time-domain signal can be transformed into the frequency domain through FFT, giving each pixel in the image its unique broadband THz signal characteristics.

### 2.2. Degradation Model

To train a model for reconstructing low-resolution (LR) images into high-resolution (HR) images, pairs of HR and LR images are needed. However, obtaining these pairs from THz images is challenging. Therefore, it is necessary to degrade HR images to create LR images that closely resemble the characteristics of THz images. This enables the training of the model using these artificially created pairs. The degradation model used in this process is of great importance. It must be effectively designed to simulate the real-world behavior of THz images to ensure the model’s performance and accuracy in practical applications.

In the context of image super-resolution, the degradation model is typically represented by the following equation:

(1) $$I_{LR}=\left(I_{HR}*k\right){↓}_{s}+n$$

where $I_{LR}$ denotes the LR image, $I_{HR}$ represents the HR image, *k* is the blurring kernel, ∗ refers to the convolution operation, ${↓}_{s}$ signifies downsampling by a scaling factor, and *n* is the additive Gaussian noise. This model describes how a high-resolution image is transformed into its low-resolution counterpart. It involves the application of a blurring kernel *k* to the high-resolution image, followed by downsampling with a scaling factor *s*, and finally the addition of noise *n*. The goal of THz image super-resolution is to restore the $I_{HR}$ from the $I_{LR}$.

The Gaussian blur kernel is often treated as the point spread function (PSF) of the THz imaging systems. The PSF depends on the imaging system and usually can be approximately an isotropic Gaussian blur kernel [7]. Given the presence of defocusing in real-world imaging and the use of a frequency-seeping source by the system, the blur kernel undergoes changes within a specific range. As a result, to enhance the model’s robustness, all conceivable blur kernels should be included in the training set. Because obtaining all the blur kernels experimentally is challenging, and THz beams usually follow the Gaussian distribution [15,16], we substitute the actual PSF with the Gaussian blur kernels. The equation is as follows:

(2) $$G\left(x,y\right)=\frac{1}{2\pi \sigma^{2}}\exp\left[-\frac{\left(x^{2}+y^{2}\right)}{2\sigma^{2}}\right],$$

where σ represents the standard deviation, which is the width of the Gaussian kernel. It is important to include multiple levels of noise in the training set, as THz waves may produce varying levels of noise when interacting with different substances.

When forming a Gaussian blur kernel, the standard deviation σ is randomly selected from a range between α and β. This can be mathematically represented as

(3) σ∼U(α,β)

where U(α,β) denotes the uniform distribution in the range. The random selection of σ introduces variability in the blurring process, which is a crucial aspect in simulating different levels of image degradation.

### 2.3. Network Architecture

**Figure 2:**
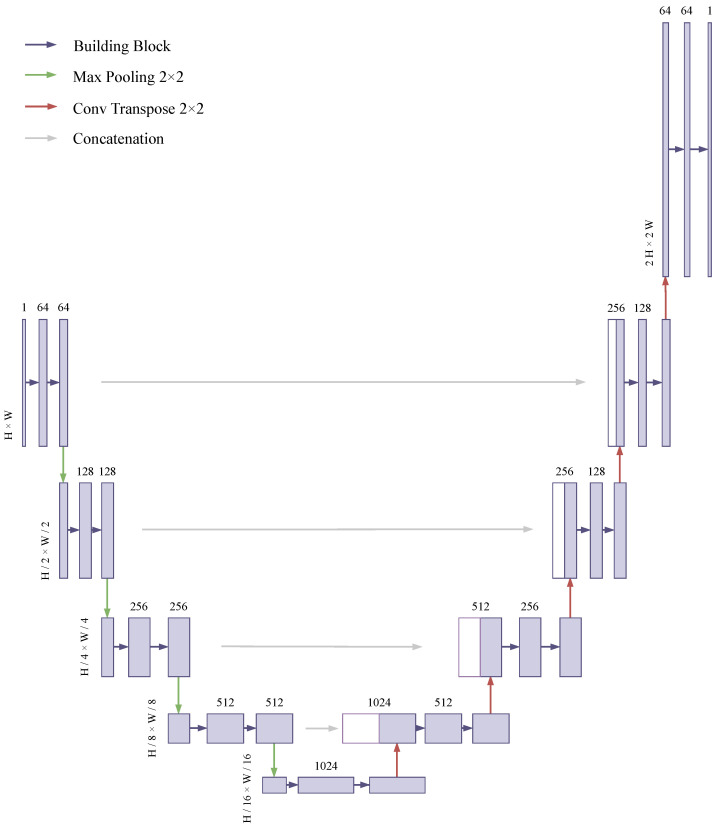
Network architecture of the proposed J-Net.

In this section, we build a novel architecture for THz image super-resolution. We modify the U-Net [9] architecture by incorporating an additional expansive path at the end. This modification is aimed at increasing the size of the feature map for super-resolution. This results in a shape that resembles the letter “J” rather than “U”, as shown in Figure 2. The original U-Net architecture is a convolutional neural network (CNN) often used for image restoration. Its structure, shaped like the letter “U”, comprises two parts: the contraction path for downsampling and the expansive path for upsampling.

1. Contraction path: This section captures contextual information from the image, utilizing convolutional layers followed by max pooling. This process reduces the image’s spatial dimensions, enabling the network to extract essential features, which is crucial for restoring degraded parts of an image.
2. Expansive path: In this part, the network upscales the feature maps to reconstruct the image at its original resolution. The use of transposed convolutions or up-convolution layers is common here. This path also involves the concatenation of feature maps from the contraction path, allowing the network to utilize both high-level and detailed information, which is critical for accurately restoring image details.
3. Skip connections: A standout feature of U-Net is its use of skip connections. These connections help transfer detailed information by linking feature maps from the contraction path directly to the expansive path. This feature is particularly beneficial in image restoration as it allows for the preservation and incorporation of fine details in the restored image.

The key differences from the basic U-Net in our approach lie in the addition of an extra expansive path towards the end and the building blocks used. We adopted the building block as the simple baseline block [10]. The details of the block are described in the following subsection.

### 2.4. Building Block

**Figure 3:**
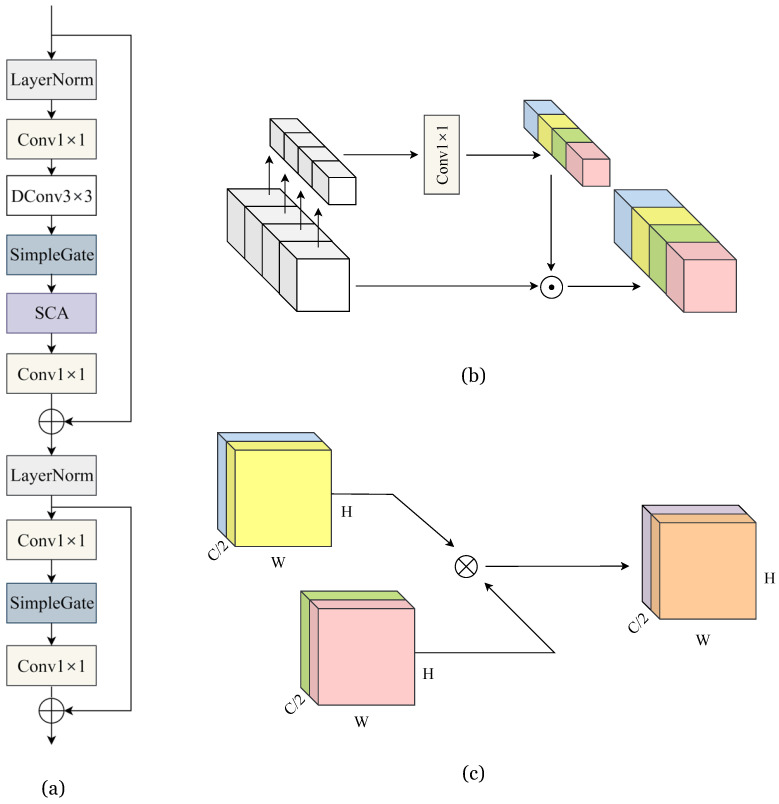
Description of building block used in J-Net. (**a**) Baseline block used in J-Net; (**b**) simplified channel attention (SCA) module; (**c**) simple gate.

The J-Net framework utilizes a simple baseline block [10] that extends beyond mere convolution layers. This essential block combines innovative components used in the latest image restoration methods. First, it incorporates layer normalization (LN) [17], which not only streamlines the block’s configuration but also stabilizes the training phase. This stability allows for an increased learning rate, boosting it from 0.0001 to 0.001, enhancing both its deblurring and noise reduction capabilities [10]. Secondly, the incorporation of channel attention in our approach enhances the computational efficiency and imparts global context to the feature maps. Thirdly, instead of using nonlinear activation functions like GELU [18], we employ a straightforward gate, which involves an element-wise multiplication of feature maps. This gate effectively mimics the role of nonlinear activation functions and contributes to improved performance. Lastly, the block is composed of layer normalization (LN), convolution, the simple gate, and a streamlined version of channel attention, termed simplified channel attention (SCA). This configuration excels in image restoration tasks, striking a remarkable balance between simplicity and efficacy. This block and its components are described in Figure 3.

## 3. Experimental Results

### 3.1. Datasets

Our research utilized a combination of the DIV2K [19] and Flickr2K [20] datasets for training the THz image super-resolution model. These datasets, integral in the image processing domain, provided a rich variety of scenarios. DIV2K, with its 1000 diverse images, including 800 for training and 200 for testing and validation, offered high-quality 2K resolution images. Flickr2K complemented this with additional versatility, allowing for the creation of datasets from training or validation sets and encompassing both low- and high-resolution images. This comprehensive approach using both datasets was crucial for effectively training our THz image super-resolution model.

**Figure 4:**
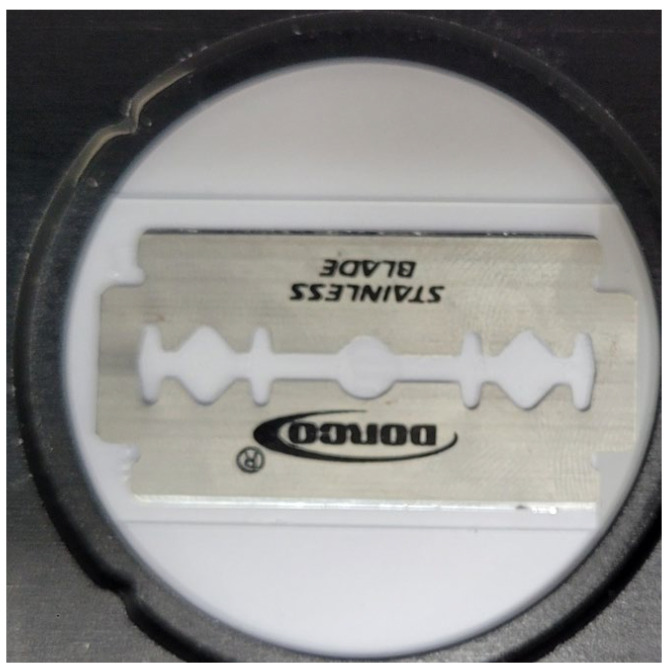
Optical image of a metal knife used for measuring THz image.

To test the proposed method, we measure a metal knife (shown in Figure 4) using a THz time-domain spectroscopy (TDS) system.

### 3.2. Implementation Details

During the training phase, we converted the RGB images to the YCbCr color space and then used only the Y channel. Additionally, we enhanced the DIV2K [19] and Flickr2K [20] datasets through data augmentation, employing random flipping and rotation with a probability of 0.5. We standardized the number of baseline blocks to 2 and maintained a uniform width of 64 in each block. The batch size was set to 32, and image patches of 96 × 96 were randomly cropped from high-resolution (HR) images for training. We initiated the training with a learning rate of $1\times 10^{-3}$, which was progressively decreased to $1\times 10^{-6}$, following a cosine annealing schedule, as per Loshchilov et al. [21]. The training was extended up to 200,000 iterations. For implementation, we used the PyTorch deep learning framework [22], and the experiments were performed using eight NVIDIA A100 GPUs. The optimization was carried out using the Adam optimizer [23], with settings $\beta_{1}=0.9$, $\beta_{2}=0.99$, and a weight decay of $1\times 10^{-4}$, targeting the minimization of the loss function:

(4) $$L_{MSE}=\frac{1}{N}\sum_{i=1}^{n}\left|\right|I_{HR}-I_{SR}{\left|\right|}^{2},$$

where *N* denotes the number of training samples, $I_{HR}$ denotes the HR image, and $I_{SR}$ represents the image predicted by a trained super-resolution (SR) model. $L_{MSE}$ denotes the mean squared error between the HR images and the predicted SR images.

### 3.3. Results and Discussion

#### 3.3.1. U-Net vs. J-Net

To show the effectiveness of the proposed J-Net, we have compared the original U-Net [9] and J-Net. In this section, naive convolution layers are used as the building blocks of the network. U-Net was originally designed for medical image semantic segmentation; however, it also achieves great performance on image restoration. So, the U-shaped network structure is widely used in the image restoration field [10,11,12,24,25,26]. Since the process of image super-resolution is one of image restoration, U-Net also could be effective for image super-resolution. We conducted experiments for three types of network architecture structures: a series of basic convolution layers followed by a PixelShuffle (PS) layer [6] called Flat U-Net; U-Net with the PS layer; and J-Net. We measured the peak signal-to-noise ratio (PSNR) on the DIV2K validation set. Table 1 shows the PSNR results on the DIV2K validation set, and indicates that J-Net is more efficient than other structures in image super-resolution.

#### 3.3.2. Variation of Degradation Parameter

**Figure 5:**
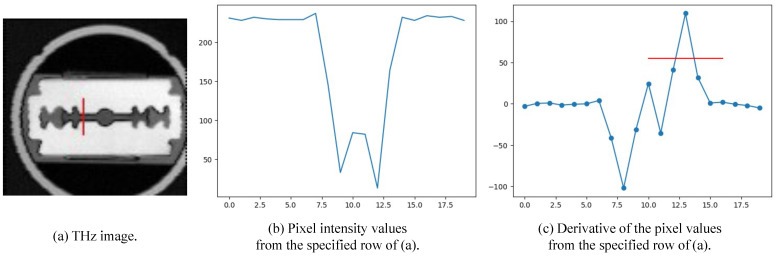
Estimated point spread function (PSF) based on the actual full width at half maximum (FWHM).

**Figure 6:**
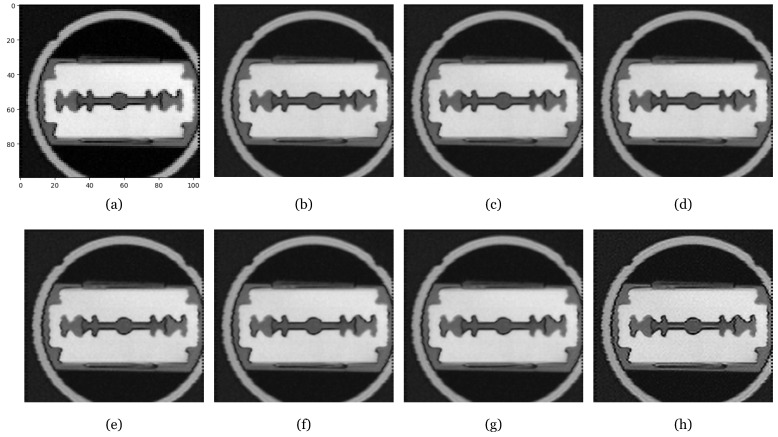
Experimental results on real THz image of metal knife. (**a**) Original THz image obtained at a frequency of 1.0 THz; (**b**) α=0,β=1; (**c**) α=0,β=3; (**d**) α=0,β=5; (**e**) α=0,β=10; (**f**) α=0.1,β=1; (**g**) α=0.1,β=3; (**h**) α=1,β=2.

We randomized the standard deviation σ value of the Gaussian blur between α and β. We show qualitative and quantitative results for different values of α and β. The range for the alpha and beta values was determined based on the actual full width at half maximum (FWHM) measurements used to estimate the point spread function (PSF), as illustrated in Figure 5. For accurate measurement of the PSF, it is necessary to examine the cross-sectional image of the object taken at a right angle. In our work, we focused on the central gap of the blade, highlighted by the red line in Figure 5a, and present the cross-sectional image of this gap in Figure 5b. Following this, Figure 5b was processed to derive Figure 5c, in which the PSF is modeled as a Gaussian distribution, enabling the estimation of its standard deviation. Although the precision of this estimation is somewhat constrained by the limited pixel count within the blade’s central gap, it still provides sufficient information to establish a range of standard deviations for use in our experiments. The estimated standard deviation value of the PSF, assumed to be a Gaussian distribution in Figure 5c, is 0.7. In Table 2, the smaller the difference between alpha and beta, the higher the PSNR value, and if the difference is the same, the PSNR is higher for higher alpha–beta values. You can see that the PSNR is significantly higher when alpha is 0.1 than when it is 0. Figure 6 shows the inference results using the model in the table: (a) is the original and the rest are the results of doubling the image resolution compared to the original image. Note the smoother boundaries compared to the original. It can be seen that the results for α=0 all tend to follow the noise of the original image, while for α=0.1 the noise is smoothed out. At an alpha of 1, the results do not seem to fully reflect the PSF of the THz system. Therefore, it is important to choose appropriate values for α and β.

#### 3.3.3. Model Comparison

**Figure 7:**
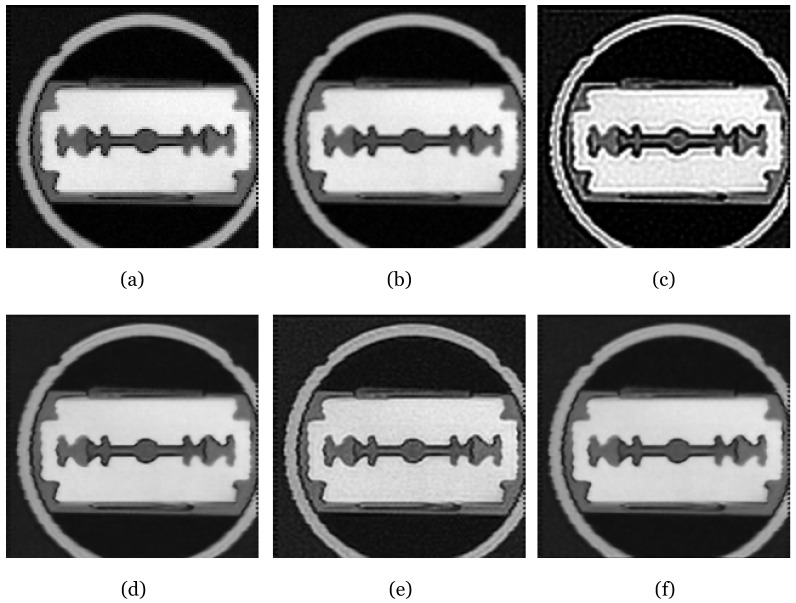
Experimental results on real THz image of metal knife. (**a**) Original THz image obtained at a frequency of 1.0 THz; (**b**) bicubic interpolation; (**c**) Lucy–Richardson deconvolution; (**d**) Long et al. [7]; (**e**) Ruan et al. [8]; (**f**) J-Net.

**Figure 8:**
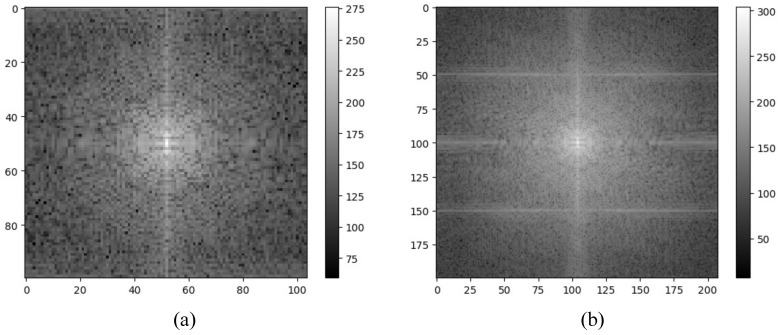
Fourier transform spectrum on real THz image of metal knife. (**a**) Original THz image obtained at a frequency of 1.0 THz; (**b**) J-Net.

We evaluate the effectiveness of our proposed method by comparing it with several established techniques, the bicubic interpolation algorithm, the widely recognized Lucy–Richardson algorithm, Long et al. [7], and U-Net, particularly focusing on real-world THz images. Since the Lucy–Richardson algorithm is primarily a deconvolution method that does not enhance image resolution, bicubic interpolation is used after its application to upscale the image. Table 3 shows that our J-Net surpasses other deep-learning-based methods [7,8] in terms of the peak signal-to-noise ratio (PSNR). The J-Net model, our proposed method, has a PSNR of 32.52 and a computational complexity of 9.29 GMac. While this complexity is notably higher than that of Ruan et al.’s method, which is at 1.39 GMac, it is significantly lower than Long et al.’s model at 126.36 GMac. This positions J-Net as a balanced approach, offering a superior PSNR with a moderate increase in computational demand, thereby providing an advantageous trade-off between performance and complexity. Moreover, Figure 7 presents a visual comparison of a THz image of a metal knife, clearly indicating that our method effectively recovers image sharpness, while the Lucy–Richardson algorithm results in distorted artifacts. Figure 8 presents the Fourier transform outcomes of the original terahertz image and the image inferred by J-Net. The original image, represented by Figure 8a, exhibits a comparatively confined central bright area with less noticeable spread of high-frequency components, indicating a resolution of average level with less emphasis on fine details and edges. In contrast, Figure 8b, depicting the inference result from J-Net, shows a significantly more extensive central bright area, especially marked along the horizontal and vertical axes by pronounced high-frequency components. This distinct distribution of high-frequency components suggests the image encompasses a richer level of detail and sharpness, thus evidencing a superior spatial resolution. Consequently, Figure 8 demonstrates that the inference result from J-Net possesses a higher spatial resolution in comparison to the original, retaining sharper edges within the terahertz imaging.

## 4. Conclusions

Our study introduces J-Net, a new neural network architecture tailored for enhancing the resolution of THz images. In comparison with traditional methods and other deep learning approaches, J-Net shows superior performance in improving image clarity, as evidenced by its higher peak signal-to-noise ratio (PSNR) in tests using the DIV2K+Flickr2K dataset and real-world THz images. The success of J-Net demonstrates its potential to significantly improve the quality of THz imaging in various applications, including security, medical imaging, and materials analysis, making it a noteworthy advancement in the field of image super-resolution.

## Figures and Tables

**Table 1:** U-Net vs. J-Net.

	Flat U-Net	U-Net	J-Net
PSNR	30.17	31.38	31.53

**Table 2:** Comparison of performance based on the values of α and β in the DIV2K validation set.

α	β	PSNR
0	1	34.96
0	3	34.41
0	5	33.13
0	10	30.09
0.1	1	35.44
0.1	3	35.32
1	2	35.08

**Table 3:** Comparison of PSNR performance with other THz image super-resolution methods.

	Long et al. [7]	Ruan et al. [8]	J-Net
PSNR	32.42	30.58	32.52
Complexity	126.36	1.39	9.29

## Data Availability

Data are contained within the article.

## Abbreviations

The following abbreviations are used in this manuscript:

THz	Terahertz
PSNR	Peak signal-to-noise ratio
